# Development and validation of the chemotherapy-induced peripheral neuropathy integrated assessment – oxaliplatin subscale: a prospective cohort study

**DOI:** 10.1186/s12885-023-11541-7

**Published:** 2023-11-14

**Authors:** Zhancheng Gu, Chen Chen, Jialin Gu, Ziwei Song, Guoli Wei, Guoxiang Cai, Qijin Shu, Lingjun Zhu, Weiyou Zhu, Haibin Deng, Sheng Li, Aifei Chen, Yue Yin, Qiulan Wu, Hongyu Zhu, Guochun Li, Anwei Dai, Jiege Huo

**Affiliations:** 1 Department of Oncology, Kunshan Hospital of Traditional Chinese Medicine, Suzhou, 215399 China; 2 The Third Clinical Medical College, Nanjing University of Chinese Medicine, Nanjing, 210046 China; 3 Department of Oncology, Yancheng TCM Hospital Affiliated to Nanjing University of Chinese Medicine, Yancheng, 224005 China; 4 Department of Oncology, Affiliated Hospital of Integrated Traditional Chinese and Western Medicine, Nanjing University of Chinese Medicine, Nanjing, 210028 China; 5 Jiangsu Province Academy of Traditional Chinese Medicine, Nanjing, 210028 China; 6 Department of Colorectal Surgery, Fudan University Shanghai Cancer Center, Shanghai, 200032 China; 7 Department of Oncology, Zhejiang Provincial Hospital of TCM, Hangzhou, 310003 China; 8 Department of Oncology, Jiangsu Province Hospital, Nanjing, 210029 China; 9 Department of Oncology, Longhua Hospital, Shanghai University of Traditional Chinese Medicine, Shanghai, 350122 China; 10 Department of Medical Oncology, Jiangsu Cancer Hospital, Nanjing, 210009 China; 11 Department of Oncology, Huaian Hospital of Traditional Chinese Medicine, Huaian, 223005 China; 12 School of Nursing, Fujian Medical University, Fuzhou, 350122 China; 13 School of Medicine & Holistic Integrative Medicine, Nanjing University of Chinese Medicine, Nanjing, 210046 China

**Keywords:** Oxaliplatin-induced peripheral neuropathy (OIPN), Assessment tool, Reliability and validity analysis, Sensitivity, Patient-reported outcome

## Abstract

**Background:**

Current chemotherapy-induced peripheral neuropathy (CIPN) assessment tools mostly have poor sensitivity and weak anti-interference, so that it is sometimes difficult to provide substantive guidance for clinical intervention. This study aimed to develop an assessment tool dedicated for oxaliplatin to address these limitations.

**Methods:**

This study screened 445 OIPN-related literatures for producing a symptom list, and developed the questionnaire module through expert supplement, item generation, content correlation analysis, pre-testing, and item improvement. The validation phase used a Chinese population-based prospective cohort study from June 2021 to July 2022. Patients were requested to complete the tested questionnaire, QLQ-CIPN20 and the CTCAE grading one day before cycles 2–6 of chemotherapy. Cronbach’s α coefficient and intraclass correlation coefficient (ICC) were calculated for the internal consistency and stability analysis, respectively. Exploratory factor analysis was conducted to investigate the construct validity. The correlations among the tested questionnaire, QLQ-CIPN20 and CTCAE were compared for the criterion validity analysis. Wilcoxon signed-rank sum test was utilized to compare the sensitivity between the tested questionnaire and QLQ-CIPN20.

**Result:**

A 20-item CIPN assessment tool named chemotherapy-induced peripheral neuropathy integrated assessment – oxaliplatin subscale (CIPNIA-OS) was developed. The validation phase included 186 patients. Cronbach's α coefficient of CIPNIA-OS was 0.764 (> 0.7), and ICC was 0.997 (between 0.9 and 1). The structure of CIPNIA-OS containing seven factors was examined. The correlation coefficient between CIPNIA-OS and CTCAE was 0.661 (95%CI 0.623 to 0.695), which was significantly higher than that between QLQ-CIPN20 and CTCAE (0.417, 95%CI 0.363 to 0.469, *p* < 0.01). Besides, the total score of CIPNIA-OS was mostly higher than QLQ-CIPN20, with an average difference of 2.189 (CI 95% 2.056 to 2.322), and the difference gradually expanded with the progress of chemotherapy (*p* < 0.05).

**Conclusion:**

This study developed an original CIPN questionnaire which was dedicated for OIPN assessment. It was a comprehensive tool that covered acute OIPN symptoms and integrated features from several proven CIPN assessment tools. The validation results supported that CIPNIA-OS had satisfactory reliability, stability, construct, criterion validity, and was more accuracy and sensitive than QLQ-CIPN20 in the evaluation of OIPN.

**Supplementary Information:**

The online version contains supplementary material available at 10.1186/s12885-023-11541-7.

## Introduction

Chemotherapy-induced peripheral neuropathy (CIPN) has always been a concern in the course of cancer therapy, as well as the main reason leading to the suspension or termination of chemotherapy [1]. Over the years, the development of intervention strategies for CIPN is not optimistic, which is not only due to the complicated underlying molecular mechanisms of different chemotherapy agents, but also limited by the lack of sensitive and targeted assessment tools to monitor the efficacy of intervention strategies [2–5].

Early assessment tools for CIPN mainly based on clinician-reported outcome (CRO), including World Health Organization (WHO) Scale [6], Eastern Cooperative Oncology Group (ECOG) Scale [7], Ajani scale [8], and National Cancer Institute-Common Terminology Criteria for Adverse Events (NCI-CTCAE) [9], which tend to underestimate the degree of neuropathy and lead to evaluation bias. Given the subjective nature of neurological symptoms, patient-reported outcome (PRO) measures are increasingly recognized as essential to comprehensively collect CIPN symptom information, and the most commonly used CIPN assessment tools in clinical practice. A series of PRO assessment tools have been produced in the last two decades, such as the Functional Assessment of Cancer Therapy/Gynecologic Oncology Group—Neurotoxicity (FACT/GOG-Ntx) subscale [10], the European Organization of Research and Treatment of Cancer (EORTC) Quality of Life Questionnaire—CIPN 20-item (QLQ-CIPN20) scale [11], the Patient Neurotoxicity Questionnaire (PNQ) [12], the Chemotherapy-Induced Peripheral Neuropathy Assessment Tool (CIPNAT) [13], the Comprehensive Assessment Scale for Chemotherapy-Induced Peripheral Neuropathy (CAS-CIPN) in Survivors of Cancer [14], and the Treatment-induced Neuropathy Assessment Scale (TNAS) [15]. PRO assessment tools have been extensively used as the primary index in CIPN-associated clinical trials, but still with some limitations in practical application. Most assessment tools attempt to be universally applicable, thus abandoning some specific neuropathic symptoms caused by individual chemotherapeutic agents, which would result in low overall-level positive response rate (O-PRR, which refers to the proportion of items with answers other than "none" or "not at all" in the overall scale). Floor effects, reflected in the low and close total score, reduces the sensitivity and anti-interference of the tools, making it susceptible to the basic disease and the subjective misjudgment of patients and difficult to reflect the change of symptoms under the intervention strategies. Therefore, developing a set of highly sensitive and targeted CIPN assessment tools could be the key of solving the problems above.

As oxaliplatin is the core agent in chemotherapy regimens such as FOLFOX, XELOX, SOX, oxaliplatin-induced peripheral neuropathy (OIPN) has become one of the most common adverse effects of the gastrointestinal cancer treatment, as well as the most noteworthy one in CIPN. OIPN can be subdivided into acute and chronic forms. About 89% of patients would experience acute OIPN, which mainly manifested as sensitivity to touching or swallowing cold items, throat discomfort, and muscle cramps, appeared within a day after the infusion of oxaliplatin, peaked in severity at day 3, and then alleviated. Chronic OIPN presents dose accumulation, mainly manifested in paresthesia symptoms including numbness and tingling at the extremities of the limbs, and a small number of proprioceptive disturbance and ataxia. Symptoms persist during treatment and can be aggravated after drug withdrawal (known as "coasting" phenomenon). The recovery is so slow that even 18 months after completing chemotherapy, 19% of patients still suffer from severe neuropathic symptoms [16]. In the early stage, acute OIPN has not received much attention as it is short-term reversible. More effort has been put into the prevention and treatment of chronic OIPN, until studies found that the condition of acute OIPN could predict the development of chronic OIPN [16–18]. Considering the refractory nature of chronic OIPN and the pervasiveness of acute OIPN, timely assessment of acute OIPN is obviously more valuable. However, existing CIPN assessment tools are inadequate for acute symptoms so that it is difficult to provide early warning for chronic progress. The purpose of this study is to develop an CIPN assessment tool dedicated for oxaliplatin to enhance the clinical practicability of the CIPN assessment tools, promote the development of CIPN precision assessment with more targeted and accurate content design, and provide support for the development of CIPN prevention and treatment strategies. In addition, we hope to further develop other CIPN assessment tools with this standardized model, so as to establish a systematic and accurate evaluation system.

## Methods

### Overall design

**Fig. 1:**
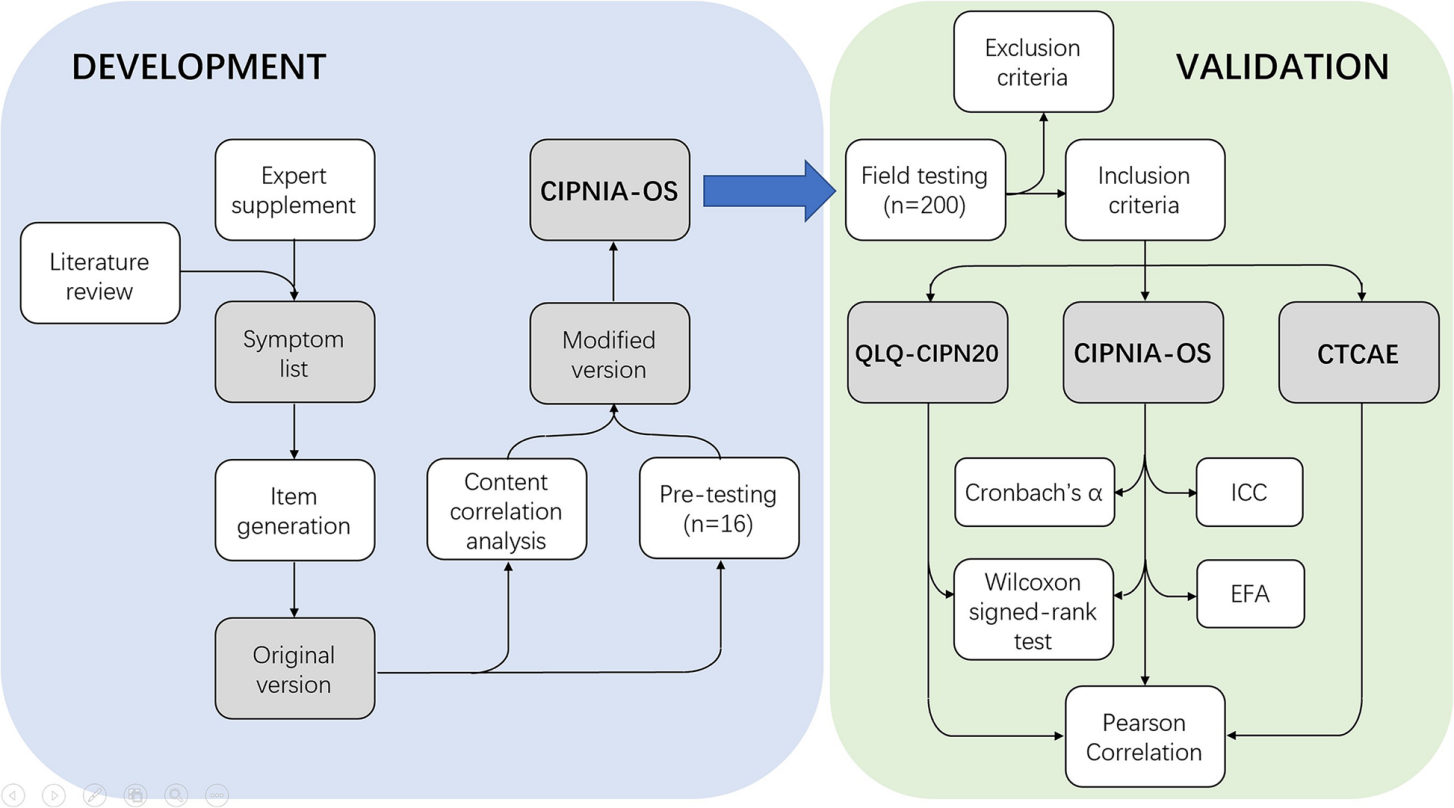
Flow chart of development and validation. CIPNIA-OS: Chemotherapy-induced Peripheral Neuropathy Integrated Assessment – Oxaliplatin Subscale; QLQ-CIPN20: European Organization of Research and Treatment of Cancer Quality of Life Questionnaire-CIPN 20-item; CTCAE: National Cancer Institute-Common Toxicity Criteria Adverse Events; ICC: Intraclass Correlation Coefficient; EFA: Exploratory factor analysis

In order to standardize the development of the questionnaire module, the EORTC questionnaire module development guidelines were used as references [19]. The development phase was separated into three steps. The first step was literature review for sorting out OIPN-related symptoms and producing a symptom list. An expert panel, consisted of nine oncologists and three nursing experts with extensive experience in OIPN prevention and treatment, was assembled for symptom supplement. The second was item generation, that is, to translate the symptoms into plain language and generate an preliminary version of the questionnaire. The third was item improvement. Items were deleted, added or reorganized according to the content correlation analysis carried out by the expert panel and the pre-test performed among a few of patients, so as to generate a modified version of the questionnaire. In the validation phase, the field test was conducted in a Chinese population-based prospective cohort of patients, and testing data were analyzed for the reliability, validity and sensitivity of the questionnaire. The flow chart is shown in Fig. 1.

## Literature review

A keyword search was conducted in Pubmed, MEDLINE and CNKI. Keywords included "oxaliplatin" or "platinum" together with "neuropathy" or "neurotoxic", and the time frame was from 2004 to 2023. Literature with no or insufficiently precise description of OIPN-related clinical symptoms was excluded. A total of 445 literatures were searched, and 22 literatures with relatively detailed descriptions of OIPN-related symptoms were retained after screening, including 10 reviews, 8 prospective clinical studies, 3 retrospective analyses, and 1 mechanism research, as shown in Supplementary Table S1 [16, 18, 20–39].

### Item generation

The expert panel put forward some suggestions according to the symptom list before drafting, which can be summarized as follows: a) medical terms need to be replaced with readily comprehensible and realistic descriptions; b) symptoms that have similar meanings or contain each other need to be merged; c) attributes of symptoms need to be more precise.

The expert panel also put forward five points of controversy. First, is it necessary to distinguish between upper and lower limbs according to the distribution of symptoms? Previous study recommended that symptoms in the upper and lower extremities should be distinguished distinctly as they are not identical [40], so it was necessary to evaluate the upper and lower limbs separately.

Second, is it necessary to distinguish between left and right limbs according to the distribution of symptoms? OIPN symptoms are mostly symmetrically distributed, and the difference between the left and right limbs, which may be caused by different habits of using the left and right limbs (for example, the right hand is more used for writing and using knives), does not affect the evaluation of the severity. In fact, patients tend to pay more attention to the more severe side, which is more valuable for the description of the severity of OIPN. So it does not need to distinguish between left and right.

Third, is it necessary to distinguish between acute and chronic symptoms? The clinical manifestations of acute and chronic OIPN are similar in many aspects, but differ only in terms of location, duration, and influence on daily life. Therefore, acute and chronic symptoms can be distinguished by adding items evaluating location, duration and influence on daily life.

Fourth, is it necessary to evaluate the attributes of each symptom (location, duration, influence on daily life)? In fact, the early symptoms of OIPN are mostly single. Even if more than two symptoms are present at the same time, attention to the most severe symptom is more valuable for evaluation.

Fifth, should objective assessment items be included, such as quantitative sensory testing (QST)? Given the objective tests had high requirements in assessing time point, skills and instrumentation or equipment, it was difficult to conduct frequent monitoring and widespread clinical application as the gold standard [41], so that they were not considered to be included.

### Content correlation analysis

The evaluation of content validity mainly adopts the content validity index (CVI) based on experts' understanding of content correlation with the evaluation object as customary, which includes the item-level content validity index (I-CVI) and the scale-level content validity index (S-CVI). Items with excellent content validity should be composed of I-CVIs of 0.78 or higher [42]. However, in the actual evaluation process, a few of experts only linked the content correlation with the clinical incidence, which led to some clinically uncommon symptoms easy to be classified as low correlation items and excluded (such as sensory ataxia, although the incidence is not high, but its impact on chemotherapy adjustment may be decisive). To avoid this, the expert panel was called upon to evaluate clinical incidence and severity of symptoms that affect the chemotherapy adjustment based on their own perceptions, thereby quantifying the rationality and importance of each item and calculating the corresponding content correlation index (CCI). The expert-cognitive clinical incidence (assume the value as “i”) was classified into four levels, including remote (i = 0.25), low (i = 0.5), moderate (i = 0.75) and high (i = 1.00), the higher the incidence, the more valuable it is for evaluation. The expert-cognitive severity of symptoms affecting chemotherapy adjustment (assume the value as “s”) was classified into three levels, including mild (s = 1.00), moderate (s = 0.66) and severe (s = 0.33), the less severe the symptoms that affect chemotherapy adjustment, the more the symptoms weigh on the evaluation. Take the average of i and s as the CCI of each item, and to ensure that the assessment was accurate and valuable, we excluded items with an CCI lower than 0.5.

### Pre-test

The pre-test was conducted in Jiangsu Province Hospital and Affiliated Hospital of Integrated Chinese and Western Medicine, Nanjing University of Chinese Medicine, both located in Nanjing, China. Sixteen colorectal cancer patients with different clinical and demographic characteristics (age, gender, educational background, chemotherapy cycle, dose of oxaliplatin, and neuropathic symptom) were selected from the two centers as subject representatives. Each subject was interviewed and the content was recorded. The interview questions included: a) Do you understand the symptoms or conditions described in the questionnaire? If not, which item or items make you confused? b) Are you willing to answer the questions truthfully? If not, what is your concern or scruple? c) Do you have any suggestion or supplement for the questionnaire? The item-level positive response rate (I-PRR, which refers to the proportion of subjects who gave the answers other than "none" or "not at all" for a single item) and the interview content would be analyzed for the basis of item improvement.

### Field test

The field test was performed among patients from 30 centers of the TIMEPOINT study from June 2021 to July 2022. The center list was shown in Supplementary Table S2. The TIMEPOINT study is an ongoing multicenter, randomized, double-blind, placebo-controlled clinical trial for exploring the efficacy of herbal prescription granules on OIPN (ClinicalTrials.gov ID: NCT04690283) [43]. Taking the time window for symptom observation into account, we set independent eligibility criteria based on the TIMEPOINT study. The inclusion criteria were as follows: a) patients with malignant tumors using oxaliplatin-based chemotherapy regimens; b) predicted interval between chemotherapy cycles ≥ 3 weeks; c) Karnofsky performance status (KPS) scale ≥ 60; and d) patients aged between 20 and 75 years. The exclusion criteria were as follows: a) patients treated with other neurotoxic non-chemotherapeutic agents; b) patients with peripheral neuropathy induced by diabetes, uremia, nervous system malignancy, spinal degeneration, limb osteoarthropathy and other causes; c) patients with Alzheimer's disease, Parkinson's disease, Huntington's disease and other neurodegenerative diseases; d) patients with dyslexia, depression, anxiety, schizophrenia and other mental diseases; e) patients with a history of hereditary/familial neuropathy; and f) other situations of inability or unwillingness to complete the evaluations. All patients were required to sign an informed consent form before being included.

The sample size was set at 200 based on the internal consistency criteria (‘Excellent’) for sample size (number of items × 7 and ≥ 100 subjects) in the COnsensus-based Standards for the selection of health Measurement INstruments (COSMIN) guidelines [44]. Considering that OIPN symptoms change gradually as chemotherapy progresses, repeated tests in the same cohort may yield different results. Therefore, we repeated the test 5 times in the same cohort of patients, with each subject completing the tested questionnaire and QLQ-CIPN20 scales and cooperating with the clinician CTCAE grading one day before cycle 2 to cycle 6 chemotherapy, so as to provide sufficient data for reliability and validity analysis and sensitivity comparison. There are two reasons to set the evaluation time one day before each cycle of chemotherapy. The first reason is to get the whole picture of OIPN over the full time period following the previous cycle of chemotherapy. Although acute OIPN usually recovers within a week, it is feasible to ask subjects to recall their condition of neuropathy within nearly a month, and it will not result in a lack of assessment of the persistent symptoms. The second reason is to enhance the operability and convenience of the assessment tool. Considering the frequent visits to the hospital during chemotherapy, it is difficult and unrealistic to require subjects to make a special trip to the hospital just to assess the degree of neuropathy. In contrast, the evaluation before each chemotherapy greatly enhances the convenience and operability of assessment. In addition, subjects were asked to complete another time of the tested questionnaire prior to infusion on the day of cycle 6 chemotherapy to provide data for the test–retest reliability analysis, and the interval from the preceding completion was ≥ 12 h.

### Statistical analysis

All analyses were conducted using SPSS Statistics Software version 27 (IBM). Cronbach's α coefficient was calculated for the internal consistency, and intraclass correlation coefficient (ICC) was calculated for the stability of the questionnaire. As a new developed questionnaire, exploratory factor analysis (EFA) was more suitable for investigating the construct validity. Before conducting EFA, Kaiser–Meyer–Olkin (KMO) measures of sampling adequacy and Bartlett's test of sphericity were used to evaluate whether it was suitability for factor analysis. Principal component analysis method was used to extract common factors, and factors with eigenvalues > 1 and variance interpretation rate ≥ 5% would be retained. Factor rotation was performed using varimax rotation to find the corresponding relationship between the factors and items, and to give reasonable factor interpretations of the tested questionnaire. CTCAE was used as the gold standard for the criterion validity analysis, and the Pearson correlation coefficients between the tested questionnaire, QLQ-CIPN20 and CTCAE were calculated and compared to evaluate the accuracy. As the two questionnaires (the tested questionnaire and QLQ-CIPN20) have the same item number and the scoring algorithm, they can be homogeneously compared. Wilcoxon signed-rank sum test was used to compare the difference in results between the test questionnaire and QLQ-CIPN20 to demonstrate sensitivity.

## Results

### Symptom list and supplement

The list of OIPN-related symptoms reported in previous literature was listed, as shown in Supplementary Table S3. Build on previous reports, the classification of OIPN as acute and chronic is justified. Acute OIPN symptoms usually include cold-sensitive paresthesia (abnormal sensation represented by tingling and numbness) and dysesthesia (hypersensitivity or pain induced by touch) in the distal extremities or the perioral region, pharyngolaryngeal dysesthesia (causing swallowing or breathing difficulty), motor dysfunction (muscular fasciculation, tetanic spasm, and prolonged contraction) and rare visual impairment. Chronic OIPN symptoms are predominantly persistent paresthesia in the distal extremities, occasionally progressing to sensory ataxia and functional impairment (causing difficulty balancing, standing, walking and manipulating small objects such as writing, fastening buttons, and holding cups) [16, 18, 20–39]. According to the discussion the expert panel, the symptom list had been basically well-rounded and no supplement was proposed. Considering that acute OIPN mainly occurs during or shortly after chemotherapy and appears to predict the development of chronic OIPN [16–18], it is feasible and valuable to be evaluated.

### Generation and improvement of the tested questionnaire

According to the suggestions put forward by the expert panel and the subject representatives, we formulated a 32-item questionnaire named "Chemotherapy-induced peripheral neuropathy Integrated Assessment – oxaliplatin subscale (preliminary version)" (CIPNIA-OS), as shown in Supplementary Fig. S1. The medical terms were concretized (for example, paresthesia was replaced by tingling and numbness), and the duplicate contents were merged (for example, cold allodynia, cold hyperalgesia, mechanical hyperalgesia, and touch evoked pain were combined into oversensitivity or pain by deleting triggers). To make symptom attributes more precise, we set up items evaluating the location, duration, and influence of the most severe symptom to the end, which were classified according to Total Neuropathy Score (TNS) [45], Levi’s scale [46], and NCI-CTCAE [9], respectively, inspired by the design of Patient-Reported Outcomes version of the CTCAE (PRO-CTCAE) [47].

Item improvement mainly depended on the results of CCI and pre-test I-PRR. The distributions of the expert-cognitive clinical incidence and severity of symptoms affecting chemotherapy adjustment of each item in the preliminary version were shown in Supplementary Fig. S2, based on which, CCIs of items were calculated and shown in Table 1. CCI less than 0.5 indicated a low content correlation, both in terms of the clinical incidence and the degree of impact on chemotherapy adjustment. Combining with the pre-test I-PRR, as shown in Supplementary Fig. S3, 8 items (items 5, 6, 12, 15, 16, 19, 20, 22) were deleted as low CCI (< 0.5) and I-PRR (= 0%). Items with I-PRR of less than 15% of the pre-test results were further interviewed to rule out accidental event. We found that stiffness of hands or arms, visual and auditory function impairment were usually present before chemotherapy, and facial cramps reported by only one patient occurred more than 3 weeks after chemotherapy and persisted just several minutes, which was considered to have a low correlation with OIPN. Therefore, items 11, 21, 28 and 29 were also considered for deletion. Compared to pain or burning sensation, hypersensitivity was reported more commonly and contained a broader description, so items 7 and 8 were revised. Besides, those patients with hand weakness were more likely to report an inability to lift or grasp objects such as cups, so item 25 was revised. Based on the improvements above, the modified version of the questionnaire containing 20 items was generated, as shown in Supplementary Fig. S4. In addition, we compared the modified version of CIPNIA-OS with current CIPN assessment tools, as shown in Supplementary Table S4.

**Table 1:** Content correlation index (CCI) of the preliminary version

Item	OIPN-related index	Item	OIPN-related index	Item	OIPN-related index
1	0.69	12	0.48^a^	23	0.76
2	0.69	13	0.51	24	0.69
3	0.71	14	0.51	25	0.63
4	0.71	15	0.40^a^	26	0.67
5	0.30^a^	16	0.40^a^	27	0.64
6	0.30^a^	17	0.69	28	0.44^a^
7	0.54	18	0.71	29	0.44^a^
8	0.54	19	0.30^a^	30	0.68
9	0.57	20	0.40^a^	31	0.68
10	0.57	21	0.40^a^	32	0.81
11	0.48^a^	22	0.49^a^		

^a^CCI < 0.5

### Clinical and demographic characteristics of the field test subjects

A total of 207 patients were included in this study. 21 patients failed to complete 6 cycles of chemotherapy during treatment and were excluded, including 9 patients whose oxaliplatin-based chemotherapy regimen was adjusted due to disease progression, and 12 patients who voluntarily terminated chemotherapy due to adverse reactions other than peripheral neuropathy (7 had severe nausea and vomiting, 3 had severe myelosuppression, 1 had severe allergic skin reactions, and 1 had unexplained discomfort). Finally, data from 186 subjects were analyzed for the reliability, validity, and sensitivity of CIPNIA-OS.

The clinical and demographic characteristics of the subjects were shown in Table 2. The average age of the subjects was 57.7 years old, and 55.91% of them were female. The educational background was mostly from secondary to undergraduate level, accounting for 89.25%. Most subjects were treated with the XELOX regimen, accounting for 96.24%. 34.41% of the subjects received a cumulative dose of oxaliplatin more than 750 mg/m^2^. The average total scores of CIPNIA-OS and QLQ-CIPN20 were 5.12 (95% CI 4.91 to 5.33) and 2.93 (95% CI 2.77 to 3.09), respectively.

**Table 2:** Clinical and demographic characteristics of subjects

Characteristics	Statistical results
Age	
Average (variance; range)	57.7 (9.76; 27–74)
Gender	
Male	82 (44.09%)
Female	104 (55.91%)
Educational background	
Primary	18 (9.68%)
Secondary	109 (58.60%)
Undergraduate	57 (30.65%)
postgraduate	2 (1.07%)
Chemotherapy regimen	
XELOX	179 (96.24%)
FOLFOX	7 (3.76%)
Cumulative dose of oxaliplatin	
< 750 mg/m^2^	122 (65.59%)
≥ 750 mg/m^2^	64 (34.41%)
CIPNIA-OS	
Average (variance; range)	5.12 (3.28; 0–19)
95% confidence interval	4.91 to 5.33
QLQ-CIPN20	
Average (variance; range)	2.93 (2.47; 0–15)
95% confidence interval	2.77 to 3.09

### Reliability analysis

The results of the reliability analysis were shown in Table 3. Cronbach's α coefficient of CIPNIA-OS was 0.764 (> 0.7), indicating that the internal consistency was considerable. Due to 13 patients failed or refused to complete the second time of CIPNIA-OS at cycle 6 chemotherapy, 173 data sets were collected and calculated for the intraclass correlation coefficients (ICC). The ICC was 0.997, close to 1, indicating that the stability of CIPNIA-OS was good.

**Table 3:** Reliability statistics

N. of items	Cronbach’s α	Intraclass Correlation Coefficient (ICC)		
20	0.764		Intraclass Correlation	95% Confidence Interval
		Single Measures	0.997	0.996 ~ 0.998
		Average Measures	0.999	0.998 ~ 0.999

### Construct validity analysis

In the construct validity analysis, we did not analyze the last three items together in order to make the contents of the items in the same dimension. The KMO coefficient was 0.694 (> 0.5), indicating strong correlation between variables. Bartlett's test of sphericity indicated that each variable was independent and the correlation matrix was factorable (χ^2^ = 5726.739, *p* < 0.05). Seven common factors were extracted by principal component analysis method, explaining 64.21% of the cumulative variance, as shown in Supplementary Table S5. Factor loading of the rotated component matrix was shown in Table 4. Factor loadings ≥ 0.40 were marked to explore item commonalities, and factor explanations were provided in Table 4.

**Table 4:** Exploratory factor analysis

Item		Rotated component matrix						
		1	2	3	4	5	6	7
**Factor 1: Paresthesia / dysesthesia of the lower limbs**								
	Tingling of feet and toes	0.807^a^	0.029	0.191	0.012	0.021	-0.029	0.089
	Numbness of feet and toes	0.733^a^	0.167	0.096	-0.044	-0.099	-0.222	0.031
	Oversensitivity or pain of feet or toes	0.677^a^	0.045	-0.062	-0.038	-0.005	0.090	-0.100
**Factor 2: Motor dysfunction of the lower limbs**								
	Difficulty walking or ascending steps	0.206	0.876^a^	0.034	0.001	-0.027	0.012	-0.011
	Difficulty standing balance	-0.143	0.717^a^	0.079	-0.084	0.045	-0.071	0.084
	Weakness of feet or legs	0.417	0.704^a^	-0.079	0.164	-0.028	0.054	-0.071
**Factor 3: Paresthesia / dysesthesia of the upper limbs**								
	Tingling of hands or fingers	0.090	-0.043	0.765^a^	0.197	0.061	-0.049	-0.019
	Numbness of hands or fingers	0.042	0.054	0.744^a^	0.131	-0.064	-0.235	-0.230
	Oversensitivity or pain of hands or fingers	0.055	0.076	0.718^a^	-0.056	0.105	0.229	0.085
**Factor 4: Motor dysfunction of the upper limbs**								
	Weakness of hands or arms	-0.034	0.019	0.003	0.864^a^	0.004	0.086	0.021
	Difficulty holding or grabbing	-0.011	0.031	0.127	0.839^a^	-0.007	0.091	-0.035
	Difficulty writing or buttoning	-0.015	-0.066	0.198	0.423^a^	-0.082	-0.322	0.018
**Factor 5: Paresthesia / dysesthesia of the perioral region**								
	Tingling around mouth	0.013	-0.039	0.086	0.002	0.834^a^	0.027	0.025
	Numbness around mouth	-0.076	0.043	0.004	-0.045	0.825^a^	-0.079	-0.058
**Factor 6: Muscle cramp of the upper limbs, pharynx and larynx**								
	Cramps of hands or arms	-0.066	-0.022	0.083	0.070	-0.039	0.657^a^	0.268
	Contraction sense in throat	-0.025	-0.024	-0.056	0.033	-0.043	0.655^a^	-0.252
**Factor 7: Muscle cramp of the lower limbs**								
	Cramps of feet or legs	-0.002	0.032	-0.105	0.000	-0.036	-0.013	0.908^a^

^a^Factor loading ≥ 0.4

### Criterion validity analysis

The Pearson correlation coefficient between CIPNIA-OS, QLQ-CIPN20 and CTCAE were calculated, as shown in Table 5. Both CIPNIA-OS and QLQ-CIPN20 were significantly correlated with CTCAE (*P* < 0.01). The Pearson correlation coefficient between CIPNIA-OS and CTCAE was 0.661, which was significantly higher than that between QLQ-CIPN20 and CTCAE (0.417, *p* < 0.01), indicating that CIPNIA-OS was closer to the evaluation results of CTCAE than QLQ-CIPN20.

**Table 5:** Pearson correlation

	Pearson correlation coefficient	95% confidence interval	Significance
CIPNIA-OS & CTCAE	0.661	0.623 ~ 0.695	*p* < 0.01
QLQ-CIPN20 & CTCAE	0.417	0.363 ~ 0.469	*p* < 0.01
CIPNIA-OS & QLQ-CIPN20	0.777	0.750 ~ 0.801	*p* < 0.01

### Sensitivity analysis

**Fig. 2:**
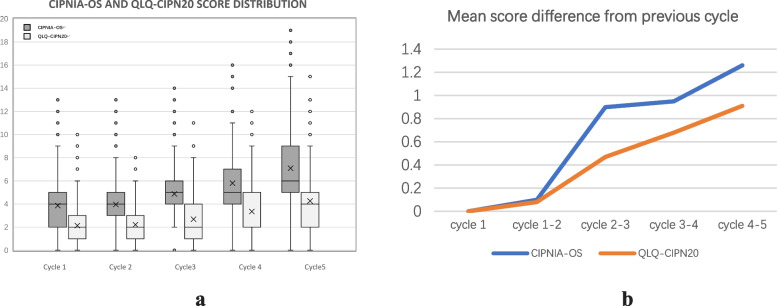
**a**. Box-plot of the score distribution of CIPNIA-OS and QLQ-CIPN20. “ × ” represents mean score, “—” represents median score, and the box represents a score range of 25% to 75%. CIPNIA-OS: Chemotherapy-induced peripheral neuropathy Integrated Assessment – oxaliplatin subscale. QLQ-CIPN20: EORTC Quality of Life Questionnaire—Chemotherapy-induced Peripheral Neuropathy 20-item. **b**. Line chart of the mean score difference of CIPNIA-OS and QLQ-CIPN20 from the previous cycle

Wilcoxon signed-rank sum test showed that, the total score of CIPNIA-OS was generally higher than that of QLQ-CIPN20 (5.12 ± 3.28 vs 2.93 ± 2.47; *p* < 0.01), and the average difference between them was 2.19 (CI 95% 2.056 to 2.322), as shown in Tables 1 and 6. Corresponding comparisons were made between them along with chemotherapy cycles, as shown in Table 7 and Fig. 2a. The mean score of CIPNIA-OS at each cycle was significantly higher than that of QLQ-CIPN20 (*p* < 0.01), and the difference gradually expanded with the progress of chemotherapy (*p* < 0.05), as shown in Fig. 2b, indicating that CIPNIA-OS had higher sensitivity.

**Table 6:** Wilcoxon signed ranks test

Ranks					Test Statistics	
		N	Mean Rank	Sum of Ranks	z	Asymp. Sig. (2-tailed)
CIPNIA-OS – QLQ-CIPN20	Negative Ranks	69^a^	325.54	22462.50	-21.164^d^	< 0.001
	Positive Ranks	722^b^	402.73	290773.50		
	Ties	139^c^				
	Total	930				

^a^ CIPNIA-OS < QLQ-CIPN20

^b^ CIPNIA-OS > QLQ-CIPN20

^c^ CIPNIA-OS = QLQ-CIPN20

^d^Based on negative ranks

**Table 7:** Score statistics of CIPNIA-OS and QLQ-CIPN20

	Cycle 2		Cycle 3		Cycle 4		Cycle 5		Cycle 6	
Mean score	3.87^a^	2.13^b^	3.97^a^	2.21^b^	4.87^a^	2.68^b^	5.82^a^	3.36^b^	7.08^a^	4.27^b^
Mean score difference from previous cycle	-	-	0.1	0.08	0.9	0.47	0.95	0.68	1.26	0.91
Mean score difference between the two tools	1.74^c^		1.76^c^		2.19^c^		2.46^c^		2.81^c^	

^a^ CIPNIA-OS

^b^ QLQ-CIPN20

^c^ CIPNIA-OS minus QLQ-CIPN20

## Discussion

The development of CIPN assessment tools has always sought to be universal, however, this is often the main reason for their low overall-level positive response rate (O-PRR) in practical application settings. Timely and precise intervention of OIPN depends on a sensitive and targeted evaluation system. Current commonly used assessment tools, whether CTCAE based on CRO [9], or QLQ-CIPN20 based on PRO [10], all have limitations such as high risk of subjective misjudgment, poor anti-interference and low sensitivity, which make them difficult to provide substantial guidance for prediction and intervention of chronic OIPN. To overcome these limitations, this study developed a CIPN assessment tool dedicated for oxaliplatin, named CIPNIA-OS, with the purpose of sensitively predicting the prognosis of OIPN and facilitating the development of a meticulous CIPN assessment system.

First of all, through literature search and expert supplement, OIPN-related symptoms were made into a list. We noted that the symptoms reported in the literature mostly contained or caused each other, for example, persistent paresthesia may be the cause of ataxia, and limb weakness may be the cause of functional impairment, so systematic induction and summary is necessary. Secondly, following the suggestions of the expert panel, symptoms were translated into easy-to-understand description items, so that a preliminary version questionnaire containing 32 items was developed, named CIPNIA-OS. With the purpose of accurately characterizing neuropathic symptom attributes, we were inspired by PRO-CTCAE [47], and designed assessment items for the location, duration, and influence of the most severe neuropathic symptoms by referring to the extent division of TNS [44], Levi’s scale [45], and NCI-CTCAE [9], respectively. This design can give more detailed tips and options and effectively reduce the risk of bias caused by subjective misjudgment of patients. Thirdly, the preliminary version was modified according to the content correlation analysis and pre-test results. Items with low CCI and I-PRR were deleted. Meanwhile, some items were revised based on subject representatives’ descriptions. Finally, a 20-item modified version was generated. What is noteworthy is that the CCI in this study is an innovation on the basis of the content validity index of the surface validity analysis.

Subsequently, the modified version was put into field testing. Data from 186 subjects were analyzed for the reliability, validity and sensitivity of CIPNIA-OS. Cronbach's α coefficient was 0.764 (> 0.7), indicating that the overall internal consistency reliability was considerable. ICC from the test–retest results was 0.997 (close to 1), supporting good reliability of CIPNIA-OS. Seven common factors were obtained in EFA, basically distributing according to the symptoms of upper and lower limbs (except perioral region). These factors explained 64.21% of the cumulative variance, more than 50%, indicating passable construct validity of the questionnaire. The total scores of CIPNIA-OS and QLQ-CIPN20 were further compared to demonstrate which of them was more superior. Firstly, correlation comparison was conducted using CTCAE as the gold standard, and the results showed that the Pearson correlation coefficient between CIPNIA-OS and CTCAE was 0.661, which was significantly higher than that between QLQ-CIPN20 and CTCAE (0.417, *p* < 0.01), indicating that CIPNIA-OS could be closer to objective evaluation results. This might be the result of CIPNIA-OS excluding items that were susceptible to underlying diseases, such as blurred vision and difficulty hearing. Secondly, Wilcoxon signed-rank sum test showed that, the total score of CIPNIA-OS was generally higher than that of QLQ-CIPN20 (5.12 ± 3.28 vs 2.93 ± 2.47; *p* < 0.01), and the average difference was 2.19 (CI 95% 2.056 to 2.322). This was apparently due to the high O-PRR of CIPNIA-OS, which made it easier to distinguish between mild and severe cases. Meanwhile, the mean score of CIPNIA-OS in each cycle was significantly higher than that of QLQ-CIPN20 (*p* < 0.01), and the difference gradually expanded with the chemotherapy cycle progressed, indicating that CIPNIA-OS had higher sensitivity.

The strengths of this study lie in the rigorous and standardized development and verification process, which were in accordance with the EORTC questionnaire module development guidelines. The questionnaire developed was original, highly targeted and sensitive. Prospective design was used in the validation phase to verify the reliability, structure, accuracy and sensitivity of the questionnaire. However, there are still some limitations. First, this assessment tool can only be used to assess the neurotoxicity of oxaliplatin, but not for other neurotoxic chemotherapeutic drugs. Second, the observation period was only limited to the chemotherapy period, and follow-up was not conducted for the post-chemotherapy phase, which may lead to the omission of some chronic OIPN symptoms. Third, all patients were hospitalized, which could potentially cause population selection bias. Fourth, although CIPNIA-OS provided some detailed features of neuropathic symptoms and improved the situation of low scoring slightly, but not ideal. Further setting up new scoring models, such as changing the weight of different items, is a possible solution. Developing specific assessment tools for different application settings is a good strategy to respond more sensitively and accurately to the actual situation. Considering that neuropathic symptoms induced by different chemotherapeutic agents have common ground, it is also promising to further develop a systematic evaluation tool with questions that can be answered selectively according to different settings.

## Conclusions

This study developed an original CIPN questionnaire, named CIPNIA-OS, which was dedicated for OIPN assessment. It was a comprehensive tool that covered acute OIPN symptoms and integrated features from several validated CIPN assessment tools. The validation results supported that CIPNIA-OS had good reliability, stability, construct, criterion validity, and was more accurate and sensitive than QLQ-CIPN20 in the evaluation of OIPN. Further study should focus on the standard of score division and the corresponding intervention strategies, as well as assessment tools for other neurotoxic chemotherapeutic agents (such as taxanes, vinca alkaloids, etc.), so as to make it systematic and really universal.

### Supplementary Information

**Additional file 1: Supplementary Table S1.** List of literature detailing OIPN-related symptoms. **Supplementary Table S2.** TIMEPOINT clinical centers in China. **Supplementary Table S3.** Previously Reported OIPN-Related Symptoms. **Supplementary Table S4.** Current assessment tools of OIPN. **Supplementary Table S5.** Total variance explained.

**Additional file 2: Supplementary Fig. S1**. Chemotherapy-induced Peripheral Neuropathy Integrated Assessment – Oxaliplatin Subscale (initial version). Scoring standard: "Not at all"=0, "A little bit"=1, "Quite a bit"=2, "Very much"=3. The symptom location was classified according to the Total Neuropathy Score (TNS). The symptom duration was classified according to the Levi’s scale. The influence was classified according to the National Cancer Institute Common Toxicity Criteria Adverse Events (NCI-CTCAE). **Supplementary Fig. S2.** Distributions of expert-cognitive clinical incidence and severity of symptoms affecting chemotherapy adjustment. The numbers in the column represent the number of experts who gave the evaluation corresponding to the color. (a) Expert-cognitive clinical incidence of oxaliplatin-induced neuropathic symptoms (scoring standard: Remote=0.25, Low=0.5, Moderate=0.75, High=1). (b) Expert-cognitive severity of oxaliplatin-induced neuropathic symptoms affecting chemotherapy adjustment (scoring standard: Mild=1, Moderate=0.66, Severe=0.33). **Supplementary Fig. S3.** Pre-testing item-level positive response rate (I-PRR) of the original version. **Supplementary Fig. S4.** Chemotherapy-induced Peripheral Neuropathy Integrated Assessment –Oxaliplatin Subscale (modified version). Scoring standard: "Not at all"=0, "A little bit"=1, "Quite a bit"=2, "Very much"=3. The symptom location was classified according to the Total Neuropathy Score (TNS). The symptom duration was classified according to the Levi’s scale. The influence was classified according to the National Cancer Institute Common Toxicity Criteria Adverse Events (NCI-CTCAE).

## Data Availability

The datasets analysed during the current study were derived from the Rinn Clinical Trial Electronic Data Acquisition System of TIMEPOINT Clinical Trials (www.rh-clinical.com). The data that support the findings of this study are available from Jiangsu Clinical Innovation Center of Digestive Cancer of Traditional Chinese Medicine but restrictions apply to the availability of these data, which were used under license for the current study, and so are not publicly available. Data are however available from the author (ZCG or JGH) reasonable request and with permission of Jiangsu Clinical Innovation Center of Digestive Cancer of Traditional Chinese Medicine.

## References

[1] Desai N, Arora N, Gupta A (2022). Chemotherapy-induced peripheral neuropathy. JAMA Intern Med.

[2] Jordan B, Margulies A, Cardoso F, Cavaletti G, Haugnes HS, Jahn P (2020). Systemic anticancer therapy-induced peripheral and central neurotoxicity: ESMO-EONS-EANO Clinical Practice Guidelines for diagnosis, prevention, treatment and follow-up. Ann Oncol.

[3] Derman BA, Davis AM (2021). Recommendations for prevention and management of chemotherapy-induced peripheral neuropathy. JAMA.

[4] Loprinzi CL, Lacchetti C, Bleeker J, Cavaletti G, Chauhan C, Hertz DL (2020). Prevention and Management of Chemotherapy-Induced Peripheral Neuropathy in Survivors of Adult Cancers: ASCO Guideline Update. J Clin Oncol.

[5] Starobova H, Vetter I (2017). Pathophysiology of Chemotherapy-Induced Peripheral Neuropathy. Front Mol Neurosci.

[6] Miller AB, Hoogstraten B, Staquet M, Winkler A (1981). Reporting results of cancer treatment. Cancer.

[7] Oken MM, Creech RH, Tormey DC, Horton J, Davis TE, McFadden ET (1982). Toxicity and response criteria of the Eastern Cooperative Oncology Group. Am J Clin Oncol.

[8] Ajani JA, Welch SR, Raber MN, Fields WS, Krakoff IH (1990). Comprehensive criteria for assessing therapy-induced toxicity. Cancer Invest.

[9] Trotti A, Colevas AD, Setser  A, Rusch V, Jaques D, Budach V (2003). CTCAE v3.0: development of a comprehensive grading system for the adverse effects of cancer treatment. Semin Radiat Oncol.

[10] Calhoun EA, Welshman EE, Chang CH, Lurain JR, Fishman DA, Hunt TL (2003). Psychometric evaluation of the Functional Assessment of Cancer Therapy/Gynecologic Oncology Group-Neurotoxicity (Fact/GOG-Ntx) questionnaire for patients receiving systemic chemotherapy. Int J Gynecol Cancer.

[11] Postma TJ, Aaronson NK, Heimans JJ, Muller MJ, Hildebrand JG, Delattre JY (2005). The development of an EORTC quality of life questionnaire to assess chemotherapy-induced peripheral neuropathy: the QLQ-CIPN20. Eur J Cancer.

[12] Hausheer FH, Schilsky RL, Bain S, Berghorn EJ, Lieberman F (2006). Diagnosis, management, and evaluation of chemotherapy-induced peripheral neuropathy. Semin Oncol.

[13] Tofthagen CS, McMillan SC, Kip KE (2011). Development and psychometric evaluation of the chemotherapy-induced peripheral neuropathy assessment tool. Cancer Nurs.

[14] Kanda K, Fujimoto K, Mochizuki R, Ishida K, Lee B (2019). Development and validation of the comprehensive assessment scale for chemotherapy-induced peripheral neuropathy in survivors of cancer. BMC Cancer.

[15] Mendoza TR, Wang XS, Williams LA, Shi Q, Vichaya EG, Dougherty PM (2015). Measuring Therapy-Induced Peripheral Neuropathy: Preliminary Development and Validation of the Treatment-Induced Neuropathy Assessment Scale. J Pain.

[16] Pachman DR, Qin R, Seisler DK, Smith EM, Beutler AS, Ta LE (2015). Clinical Course of Oxaliplatin-Induced Neuropathy: Results From the Randomized Phase III Trial N08CB (Alliance). J Clin Oncol.

[17] Park SB, Goldstein D, Lin CS, Krishnan AV, Friedlander ML, Kiernan MC (2009). Acute abnormalities of sensory nerve function associated with oxaliplatin-induced neurotoxicity. J Clin Oncol.

[18] Argyriou AA, Cavaletti G, Briani C, Velasco R, Bruna J, Campagnolo M (2013). Clinical pattern and associations of oxaliplatin acute neurotoxicity: a prospective study in 170 patients with colorectal cancer. Cancer.

[19] Sprangers MA, Cull A, Groenvold M, Bjordal K, Blazeby J, Aaronson NK (1998). The European Organization for Research and Treatment of Cancer approach to developing questionnaire modules: an update and overview. EORTC Quality of Life Study Group. Qual Life Res.

[20] Kokotis P, Schmelz M, Kostouros E, Karandreas N, Dimopoulos MA (2016). Oxaliplatin-Induced Neuropathy: A Long-Term Clinical and Neurophysiologic Follow-Up Study. Clin Colorectal Cancer.

[21] Reddy SM, Vergo MT, Paice JA, Kwon N, Helenowski IB, Benson AB (2016). Quantitative Sensory Testing at Baseline and During Cycle 1 Oxaliplatin Infusion Detects Subclinical Peripheral Neuropathy and Predicts Clinically Overt Chronic Neuropathy in Gastrointestinal Malignancies. Clin Colorectal Cancer.

[22] Binder A, Stengel M, Maag R, Wasner G, Schoch R, Moosig F (2007). Pain in oxaliplatin-induced neuropathy–sensitisation in the peripheral and central nociceptive system. Eur J Cancer.

[23] Matsumoto Y, Yoshida Y, Kiba S, Yamashiro S, Nogami H, Ohashi N (2020). Acute chemotherapy-induced peripheral neuropathy due to oxaliplatin administration without cold stimulation. Support Care Cancer.

[24] van Haren FGAM, Steegers MAH, Thijssen M, van der Wal SEI, Vissers KCP, Engels Y (2021). Qualitative Evaluation of the Influence of Acute Oxaliplatin-Induced Peripheral Neuropathy on Quality of Life and Activities of Daily Life. Pain Pract.

[25] Hsu HT, Wu LM, Lin PC, Juan CH, Huang YY, Chou PL (2020). Emotional distress and quality of life during folinic acid, fluorouracil, and oxaliplatin in colorectal cancer patients with and without chemotherapy-induced peripheral neuropathy: A cross-sectional study. Medicine (Baltimore).

[26] Yang Y, Zhao B, Gao X, Sun J, Ye J, Li J (2021). Targeting strategies for oxaliplatin-induced peripheral neuropathy: clinical syndrome, molecular basis, and drug development. J Exp Clin Cancer Res.

[27] Cavaletti G, Marmiroli P (2020). Management of Oxaliplatin-Induced Peripheral Sensory Neuropathy. Cancers (Basel).

[28] Kang L, Tian Y, Xu S, Chen H (2021). Oxaliplatin-induced peripheral neuropathy: clinical features, mechanisms, prevention and treatment. J Neurol.

[29] Wei G, Gu Z, Gu J, Yu J, Huang X, Qin F (2021). Platinum accumulation in oxaliplatin-induced peripheral neuropathy. J Peripher Nerv Syst.

[30] Sereno M, Gutiérrez-Gutiérrez G, Gómez-Raposo C, López-Gómez M, Merino-Salvador M, Tébar FZ (2014). Oxaliplatin induced-neuropathy in digestive tumors. Crit Rev Oncol Hematol.

[31] Sałat K (2020). Chemotherapy-induced peripheral neuropathy-part 2: focus on the prevention of oxaliplatin-induced neurotoxicity. Pharmacol Rep.

[32] Cersosimo RJ (2005). Oxaliplatin-associated neuropathy: a review. Ann Pharmacother.

[33] Zedan AH, Hansen TF, FexSvenningsen A, Vilholm OJ (2014). Oxaliplatin-induced neuropathy in colorectal cancer: many questions with few answers. Clin Colorectal Cancer.

[34] Egashira N (2021). Pathological Mechanisms and Preventive Strategies of Oxaliplatin-Induced Peripheral Neuropathy. Front Pain Res (Lausanne).

[35] Zahrieh D, Satele D, Smith EML, Loprinzi CL, Le-Rademacher J (2022). Temporal, Location- and Symptom-Specific Likelihood of Patient-Reported Sensory Symptoms Related to Oxaliplatin-Induced Peripheral Neuropathy (OIPN) in Patients Receiving Oxaliplatin for Three Months. Cancers (Basel).

[36] Tanishima H, Tominaga T, Kimura M, Maeda T, Shirai Y, Horiuchi T (2017). Hyperacute peripheral neuropathy is a predictor of oxaliplatin-induced persistent peripheral neuropathy. Support Care Cancer.

[37] Miura Y, Ando M, Yamazaki K, Hironaka S, Boku N, Muro K (2021). Time-dependent discrepancies between physician-assessed and patient-reported oxaliplatin-induced peripheral neuropathy in patients with metastatic colorectal cancer who received mFOLFOX6 plus bevacizumab: a post hoc analysis (WJOG4407GSS2). Support Care Cancer.

[38] Potenzieri A, Riva B, Rigolio R, Chiorazzi A, Pozzi E, Ballarini E, Cavaletti G, Genazzani AA (2020). Oxaliplatin-induced neuropathy occurs through impairment of haemoglobin proton buffering and is reversed by carbonic anhydrase inhibitors. Pain.

[39] Pasetto LM, D'Andrea MR, Rossi E, Monfardini S (2006). Oxaliplatin-related neurotoxicity: how and why?. Crit Rev Oncol Hematol.

[40] Wolf SL, Barton DL, Qin R, Wos EJ, Sloan JA, Liu H (2012). The relationship between numbness, tingling, and shooting/burning pain in patients with chemotherapy-induced peripheral neuropathy (CIPN) as measured by the EORTC QLQ-CIPN20 instrument, N06CA. Support Care Cancer.

[41] McCrary JM, Goldstein D, Boyle F, Cox K, Grimison P, Kiernan MC (2017). Optimal clinical assessment strategies for chemotherapy-induced peripheral neuropathy (CIPN): a systematic review and Delphi survey. Support Care Cancer.

[42] Polit DF, Beck CT (2006). The content validity index: are you sure you know what's being reported? Critique and recommendations. Res Nurs Health.

[43] Gu Z, Wei G, Zhu L, Zhu L, Hu J, Li Q (2021). Preventive Efficacy and Safety of Yiqi-Wenjing-Fang Granules on Oxaliplatin-Induced Peripheral Neuropathy: A Protocol for a Randomized, Double-Blind, Placebo-Controlled, Multicenter Trial. Evid Based Complement Alternat Med.

[44] Prinsen CA, Vohra S, Rose MR, Boers M, Tugwell P, Clarke M (2016). How to select outcome measurement instruments for outcomes included in a "Core Outcome Set" - a practical guideline. Trials.

[45] Cornblath DR, Chaudhry V, Carter K, Lee D, Seysedadr M, Miernicki M (1999). Total neuropathy score: validation and reliability study. Neurology.

[46] Lévi F, Misset JL, Brienza S, Adam R, Metzger G, Itzakhi M, et al. A chronopharmacologic phase II clinical trial with 5-fluorouracil, folinic acid, and oxaliplatin using an ambulatory multichannel programmable pump. High antitumor effectiveness against metastatic colorectal cancer. Cancer. 1992;69(4):893–900. 10.1002/1097-0142(19920215)69:4%3C893::AID-CNCR2820690410%3E3.0.CO;2-X. 1735081

[47] Basch E, Becker C, Rogak LJ, Schrag D, Reeve BB, Spears P (2021). Composite grading algorithm for the National Cancer Institute's Patient-Reported Outcomes version of the Common Terminology Criteria for Adverse Events (PRO-CTCAE). Clin Trials.

