# Supplementary material for: Development and validation of the chemotherapy-induced peripheral neuropathy integrated assessment – oxaliplatin subscale: a prospective cohort study
Source: BMC Cancer. 2023 Nov 14;23:1109. doi: 10.1186/s12885-023-11541-7 (PMC10648311; doi:10.1186/s12885-023-11541-7)
Supplement: Supplementary file 1 — Additional file 1: Supplementary Table S1. List of literature detailing OIPN-related symptoms. Supplementary Table S2. TIMEPOINT clinical centers in China. Supplementary Table S3. Previously Reported OIPN-Related Symptoms. Supplementary Table S4. Current assessment tools of OIPN. Supplementary Table S5. Total variance explained. [file 12885_2023_11541_MOESM1_ESM.docx]

**Development and Validation of** **the Chemotherapy-induced Peripheral Neuropathy Integrated Assessment–Oxaliplatin Subscale**

| **Supplementary Table S1. List of literature detailing OIPN-related symptoms** | | | | | |
| --- | --- | --- | --- | --- | --- |
| **No.** | **Authors** | **Study type** | **Publication time** | **Description of acute OIPN symptoms** | **Description of chronic OIPN symptoms** |
| 1 | Binder A, et al ^[22]^ | prospective clinical study | December 2007 | paraesthesia, dysaesthesia or pain;  cold and mechanical hyperalgesia;  heat hyperalgesia and warm hypoesthesia;  cold evoked pain (pain evoked by cold environmental temperature or contact with cold objects, fluids or air);  spontaneous pain in both hands;  the most frequently used pain descriptors: tingling, cold, freezing, pricking, heavy, piercing. | loss of sensation, dysaesthesias and functional impairment. |
| 2 | Argyriou AA, et al ^[18]^ | prospective clinical study | January 2013 | cold-induced perioral paresthesias;  cold-induced pharyngolaryngeal dysesthesia;  shortness of breath;  difficulty swallowing;  laryngospasm;  muscle cramps;  jaw stiffness;  visible fasciculations;  voice changes;  ptosis;  ocular changes. | sensory, axonal neuropathy with a stocking-and-glove distribution. |
| 3 | Pachman DR, et al ^[16]^ | prospective clinical study | October 2015 | sensitivity to touching cold, discomfort swallowing cold liquids, and throat discomfort;  muscle cramps. | tingling, numbness, pain. |
| 4 | Reddy SM, et al ^[21]^ | prospective clinical study | March 2016 | cold-induced dysesthesias and parasthesias of the upper extremities and face, cold “hypersensitivity”, jaw tightness, pharyngolaryngeal dysesthesia (loss of sensation of breathing without any objective evidence of respiratory distress), muscle spasms, fasciculations, voice changes, and ocular pain. | peripheral “stocking and glove” pattern;  axonal sensory neuropathy. |
| 5 | Kokotis P, et al ^[20]^ | prospective clinical study | September 2016 | cold allodynia in the distal extremities, mouth, and throat and usually associated with muscle cramps;  cold hyperesthesia and dysesthesia. | distal paresthesia and numbness;  paresthesia, dysesthesia, and hypoesthesia of the extremities. |
| 6 | Hsu HT, et al ^[25]^ | prospective clinical study | February 2020 | numbness, tingling, coldness-induced paresthesia of the peripheral limbs, paresthesia around the mouth, difficulty swallowing while drinking cold water, shortness of breath, numbness, cramps, jaw stiffness, and changes in auditory and visual receptive fields. | sensory loss, reduction of deep tendon reflexes, and abnormal proprioception;  coldness-related burning sensation or discomfort in the upper limbs, numbness in the upper limbs, tingling in the upper limbs, impairment of vision, and discomfort in the throat;  interference with activities of daily life, including fastening buttons, holding cups, using chopsticks, opening jars, writing, turning pages, holding pots and pans, and walking. |
| 7 | Matsumoto Y, et al ^[23]^ | prospective clinical study | November 2020 | dysesthesia and paresthesia of the hands, feet, and perioral region;  intense pain induced by cold wind blowing on the face or the ingestion of cold drinks | No description |
| 8 | van Haren FGAM, et al ^[24]^ | prospective clinical study | June 2021 | paresthesia triggered by cold and dysesthesias in the perioral region, hands, and feet;  tingling sensation and pain when touching anything cold;  painful, tingling feeling in the arm;  stiffness of the arm;  tingling and irritated in fingers. | persistent peripheral sensory neuropathy with a stocking-and-glove distribution. |
| 9 | Cersosimo RJ, et al ^[32]^ | review | January 2005 | paresthesia, hypoesthesia, and dysesthesia, begin in the hands or feet, also occur around the mouth or in the throat;  shortness of breath or difficulty swallowing, but without bronchospasm, laryngospasm, wheezing, or stridor;  unusual sensation in the tongue, jaw spasms, eye pain, and muscle spasms or cramps, stiffness in the hands or feet or the inability to release the grip, a feeling of pressure in the chest. | paresthesia, hypoesthesia, dysesthesia, and changes in proprioception;  changes in proprioception affect normal daily activities that require fine motor coordination such as writing, holding objects, picking up coins, and buttoning shirts. |
| 10 | Pasetto LM, et al ^[39]^ | review | August 2006 | paresthesias and dysesthesia of the hands, feet and peroral region, with jaw tightness;  pharyngo-laryngo-dysesthesia accompanied by a sensation of shortness of breath. | sensory ataxia, functional impairment, jaw pain, eye pain, ptosis, leg cramps and visual and voice changes. |
| 11 | Sereno M, et al ^[30]^ | review | January 2014 | paresthesias and/or dysesthesias in the distal extremities and/or the perioral region;  tetanic spasms, fasiculations, and prolonged muscular contractions. | dysesthesias and paresthesias of the extremities non-cold related. |
| 12 | Zedan AH, et al ^[33]^ | review | June 2014 | paresthesias (tickling or tingling sensations in the skin, whether distal or perioral), dysesthesias (abnormal sensations of touch often associated with pain);  pharyngolaryngeal dysesthesia causing a feeling of difficulty in breathing or swallowing;  muscular contractions（described as “spasms” or “cramps,” stiffness of the hands or feet, or inability to release grip）. | predominantly sensory dysfunction with distal paresthesias, often progressing to sensory ataxia and functional impairment. |
| 13 | Sałat K ^[31]^ | review | June 2020 | dysesthesia and paresthesia of the hands and feet, exacerbated by cold;  tetanic spasms, fasciculations, prolonged muscular contractions. | paresthesia, hypoesthesia and dysesthesia of the hands and feet, changed proprioception, affecting normal daily activities that require fine motor coordination. |
| 14 | Cavaletti G, et al ^[27]^ | review | May 2020 | cold-induced paresthesias, with predominant oropharyngeal, hands, and feet distribution. | numbness and tingling in hands and feet, with a distal-to-proximal extension of symptoms after increasing exposition to OHP;  difficulty in manipulating small objects (particularly if not looking at them), standing unless base widening. |
| 15 | Kang L, et al ^[28]^ | review | September 2021 | cold-sensitive peripheral paresthesia (the presence of tingling, numbness, pressure, cold or warm abnormal skin sensations), starts from the limbs, and sometimes occurs around the mouth or in the throat;  experience unusual sensations in the tongue, jaw, eyes, and muscles, associating with muscular fasciculation, tetanic spasm, and prolonged contraction. | paresthesia, sensation loss and changes in proprioception that affect daily activities, including writing, taking, and some other activities requiring fine motor coordination. |
| 16 | Wei G, et al ^[29]^ | review | March 2021 | sensory abnormalities related to cold stimuli occurring in the distal extremities;  discomfort in the oral cavity, throat, jaw, and muscle spasm. | bilateral symmetric paresthesia, dysesthesia and pain, mainly on both feet and/or at the ends of both hands. |
| 17 | Yang Y, et al ^[26]^ | review | October 2021 | triggered mainly by cold stimulation, cold-sensitive peripheral paresthesia (hands and feet dysesthesia and paresthesia) and motor symptoms (e.g., prolonged muscular contractions, tetanic spasms, and fasciculations). | sensation loss and changes in proprioception. |
| 18 | Egashira N, et al ^[34]^ | review | December 2021 | paresthesia around the limbs, mouth, pharynx, and larynx and slight motor dysfunction. | persistent numbness, paresthesias, and pain in the limbs, difficult to write letters, hold chopsticks, press buttons, or even walk. |
| 19 | Tanishima H, et al ^[36]^ | Retrospective analysis | May 2017 | perioral paresthesia, pharyngolaryngeal dysesthesia, difficult breathing and swallowing, muscle cramps, and paresthesia, and dysesthesias in the distal extremities. | noncold-induced dysesthesias and paresthesias of the distal extremities. |
| 20 | Miura Y, et al ^[37]^ | Retrospective analysis | July 2021 | perioral or pharyngolaryngeal dysesthesia, jaw stiffness, or hand dysesthesia induced by touching cold objects. | tingling, numbness, and pain, typically in the hands and feet. |
| 21 | Zahrieh D, et al ^[35]^ | Retrospective analysis | February 2022 | numbness, tingling and shooting/burning pain. | |
| 22 | Potenzieri A, et al ^[38]^ | Mechanism research | February 2020 | cramps and paresthesias/dysesthesias. | dysesthesias/paresthesias, as well as burning and lancinating pain that spreads from toes and fingers with a stocking-and-glove distribution. |

| **Supplementary Table S2. TIMEPOINT clinical centers in China** | | | |
| --- | --- | --- | --- |
| **No.** | **Organization name** | **Location** | **Investigator** |
| 1 | Jiangsu Province Hospital on Integration of Chinese and Western Medicine * | Nanjing, Jiangsu Province | Jiege Huo |
| 2 | Sir Run Run Hospital | Nanjing, Jiangsu Province | Fubing Wu |
| 3 | Yancheng Hospital of Traditional Chinese Medicine | Yancheng, Jiangsu Province | Jianlin Xu |
| 4 | Jiangning District Hospital of Traditional Chinese Medicine | Nanjing, Jiangsu Province | Zhen Wang |
| 5 | Suzhou Hospital of Traditional Chinese Medicine | Suzhou, Jiangsu Province | Min Liu |
| 6 | Huaian Hospital of Traditional Chinese Medicine | Huaian, Jiangsu Province | Aifei Chen |
| 7 | Zhenjiang Hospital of Chinese Traditional and Western Medicine | Zhenjiang, Jiangsu Province | Dong Fang |
| 8 | Changshu NO.1 People’s Hospital | Changshu, Jiangsu Province | Hong Lu |
| 9 | Affiliated Nanjing Jiangbei People's Hospital of Nantong University | Nanjing, Jiangsu Province | Ge Feng |
| 10 | The First People's Hospital of Chuzhou | Chuzhou, Anhui Province | Aimin Chen |
| 11 | Shanghai Changzheng Hospital | Shanghai | Xiaoqiang Yue |
| 12 | Nantong Tumor Hospital | Nantong, Jiangsu Province | Chunming Xu |
| 13 | Danyang Hospital of Traditional Chinese Medicine | Danyang, Jiangsu Province | Guofang Wang |
| 14 | Nanjing Drum Tower Hospital | Nanjing, Jiangsu Province | Xiaoping Qian |
| 15 | Jiangsu Cancer Hospital | Nanjing, Jiangsu Province | Liangjun Zhu |
| 16 | Jiangsu Province Hospital * | Nanjing, Jiangsu Province | Lingjun Zhu |
| 17 | Fudan University Shanghai Cancer Center | Shanghai | Guoxiang Cai |
| 18 | Longhua Hospital Shanghai University of Traditional Chinese Medicine | Shanghai | Zhongqi Wang |
| 19 | Changhai Hospital | Shanghai | Xiaofeng Zhai |
| 20 | Shuguang Hospital Affiliated to Shanghai University of Traditional Chinese Medicine | Shanghai | Qi Li |
| 21 | Yangzhou Hospital of Traditional Chinese Medicine | Yangzhou, Jiangsu Province | Xiaochun Zhang |
| 22 | Zhejiang Provincial Hospital of Chinese Medicine | Hangzhou, Zhejiang Province | Qijin Shu |
| 23 | Hangzhou Hospital of Traditional Chinese Medicine | Hangzhou, Zhejiang Province | Shengyou Lin |
| 24 | The First Affiliated Hospital of Guangxi University of Chinese Medicine | Nanning, Guangxi Province | Zhen Rong |
| 25 | Henan Cancer Hospital | Zhengzhou, Henan Province | Huaimin Liu |
| 26 | Affiliated Hospital of Traditional Chinese Medicine of Southwest Medical University | Luzhou, Sichuan Province | Zhongming Yang |
| 27 | Affiliated Hospital of Shanxi University of Traditional Chinese Medicine | Xianyang, Shanxi Province | Renting Li |
| 28 | Hunan Academy of Traditional Chinese Medicine Affiliated Hospital | Changsha, Hunan Province | Puhua Zeng |
| 29 | Hebei General Hospital | Shijiazhuang, Hebei Province | Qingxia Li |
| 30 | The First Affiliated Hospital of Guizhou University of Chinese Medicine | Guiyang, Guizhou Province | Xindong Tang |
| * Pre-test implementation Center | | | |

| **Supplementary Table S3. Previously Reported OIPN-Related Symptoms** | | | | | |
| --- | --- | --- | --- | --- | --- |
| **Acute OIPN** | | | | | |
| **Symptom** | **Location** | **Literature** | **Symptom** | **Location** | **Literature** |
| paresthesia | distal extremities  perioral region  pharynx and larynx | [18] [21] [22] [23] [24] [25] [26] [27] [28] [29] [30] [31] [32] [33] [34] [36] [37] [38] [39] | muscle cramp / muscle spasm | distal extremities  perioral region  pharynx and larynx | [16] [18] [20] [25] [27] [32] [33] [38]/ [21] [29] |
| dysesthesia | distal extremities  perioral region  pharynx and larynx | [18] [20] [21] [22] [23] [24] [30] [31] [32] [33] [36] [38] [39] | prolonged contraction / muscular contraction | distal extremities  perioral region  pharynx and larynx | [26] [28] [30] [31] / [33] |
| hypoesthesia | distal extremities  perioral region  pharynx and larynx | [22] [32] | tetanic spasm | distal extremities  perioral region  pharynx and larynx | [21] [26] [28] [30] [31] |
| tingling / pricking | distal extremities  perioral region  pharynx and larynx | [22] [24] [25] [28] [33] / [22] | muscular fasciculation | distal extremities  tongue  jaw  eye | [18] [21] [26] [27] [28] [30] [31] |
| numbness | distal extremities  perioral region  pharynx and larynx | [25] [28] | muscle stiffness | distal extremities  arms  jaw | [18] [21] [24] [25] [32] [33] [37] [39] |
| pressure | distal extremities  perioral region  pharynx and larynx | [28] [32] | weakness | [lower limbs](javascript:;) | [24] [25] |
| tickling | distal extremities  perioral region | [33] | difficulty breathing or swallowing | pharynx and larynx | [16] [18] [21] [25] [32] [33] [36] [39] |
| cold hyperesthesia / cold hypersensitivity | distal extremities  perioral region  pharynx and larynx | [20] [23] [29] / [16] [21] [26] | throat discomfort | pharynx and larynx | [16] [24] [25] [29] [39] |
| cold allodynia / cold evoked pain / cold hyperalgesia | distal extremities  perioral region  pharynx and larynx  eye | [20] / [22] [23] [25] [32] [33] / [22] | voice changes | pharynx and larynx | [18] [21] [39] |
| pain / burning pain | distal extremities  perioral region  eye | [18] [21] [22] [24] / [25] | impairment of vision | visual receptive fields | [25] [39] |
| mechanical hyperalgesia / touch evoked pain | distal extremities | [22] / [33] | impairment of hearing | auditory receptive fields | [25] |
| **Chronic OIPN** | | | | | |
| **Symptom** | **Location** | **Literature** | **Symptom** | **Location** | **Literature** |
| paresthesia | distal extremities | [20] [25] [28] [29] [30] [31] [32] [33] [34] [36] | sensory ataxia | distal extremities | [33] [39] |
| dysesthesia | distal extremities | [20] [22] [29] [30] [31] [32] [36] | changed proprioception / abnormal proprioception | proprioception | [25] [26] [27] [28] [31] [32] |
| hypoesthesia | distal extremities | [31] [32] | fine motor coordination disorder | proprioception | [28] [31] [32] |
| tingling | distal extremities | [16] [25] [27] [35] [37] | functional impairment | proprioception | [22] [33] [39] |
| numbness | distal extremities | [16] [20] [25] [27] [35] [37] | difficulty manipulating small objects | proprioception | [25] [27] [28] [31] [32] [34] |
| sensation loss | distal extremities | [20] [22] [25] [26] [28] [31] [34] | difficulty balancing | proprioception | [25] [27] |
| pain / burning pain | distal extremities | [16] [29] [34] [35] [37] [39] / [25] [38] | difficulty standing or walking | proprioception | [25] [27] [32] |

| **Supplementary Table S4. Current assessment tools of OIPN** | | | | | | | | | | | | |
| --- | --- | --- | --- | --- | --- | --- | --- | --- | --- | --- | --- | --- |
| **Tool abbreviation** | **Development or release time** | **Item count** | **Item category** | | | | | | | | | **Feature** |
|  |  |  | **Sensory function**  (content / proportion) | | | | **Motor function**  (content / proportion) | | | **Autonomic function**  (content / proportion) | **Other**  (content / proportion) |  |
| **CIPNIA-OS** | 2023 | 17+3 | Paresthesia of limbs (tingling, numbness, oversensitivity or pain) | Paresthesia around mouth (tingling, numbness) | Paresthesia in throat (contraction sense) | Deep paresthesia of lower limbs (difficulty standing balance) | Muscle spasm of limbs | Muscle weakness of limbs (weakness, difficulty holding or grabbing, difficulty walking or ascending steps) | Fine motor coordination dysfunction (difficulty writing or buttoning) | \ | \ | Likert 0-3, containing symptom attribute (location, duration, influence on daily life of chief symptom) |
|  |  |  | 35.3% | 11.8% | 5.9% | 5.9% | 11.8% | 23.5% | 5.9% | \ | \ |  |
| **FACT/GOG-Ntx** | 2003 | 13 | Paresthesia of limbs (numbness or tingling, discomfort, joint pain, pain when exposed to cold temperatures) | Impairment of hearing (trouble hearing, ringing or buzzing in ears) | Paresthesia in throat (difficulty breathing when exposed to cold temperatures) | Astereognosis (trouble feeling the shape of small objects) | Muscle spasm of limbs | Muscle weakness of limbs (feel weak, trouble walking) | Fine motor coordination dysfunction (trouble buttoning buttons) | \ | \ | Likert 0-4, described in the first person, easy to understand |
|  |  |  | 38.5% | 15.4% | 7.7% | 7.7% | 7.7% | 15.4% | 7.7% | \ | \ |  |
| **QLQ-CIPN20** | 2005 | 20 | Paresthesia of limbs (tingling, numbness, shooting or burning pain) | Impairment of hearing (difficulty hearing) | Thermesthesia dysfunction (difficulty distinguishing between hot and cold water) | Deep paresthesia of lower limbs (difficulty feeling the ground) | Muscle spasm of limbs | Muscle weakness of limbs (difficulty holding a pen, difficulty opening a jar or bottle, difficulty walking, difficulty climbing stairs or getting up out of a chair, difficulty using the pedals) | Fine motor coordination dysfunction (difficulty manipulating small objects) | Autonomic dysfunction (dizzy when standing up, blurred version, erectile dysfunction) | \ | Likert 1-4, in the form of questions, easy to understand |
|  |  |  | 30% | 5% | 5% | 5% | 10% | 25% | 5% | 15% | \ |  |
| **PNQ *Oxaliplatin*** | 2006 | 2+28 | Paresthesia of limbs (numbness, pain, burning, tingling or change of touching sense) | Paresthesia around mouth (numbness, pain, burning, tingling or change of touching sense) | \ | \ | Muscle spasm of limbs, mouth or jaw (difficulty breathing, swallowing, drinking or chewing food) | Muscle weakness of limbs (difficulty opening doors, walking, climbing stairs, driving, typing on a keyboard) | Fine motor coordination dysfunction (difficulty buttoning, zippering, fastening, writing, sewing, knitting, dialing, tying shoes, putting on jewelry, using utensils) | \ | Impact on daily life (difficulty sleeping) | Rating A-E, full coverage of the impact on daily life |
|  |  |  | \ | \ | \ | \ | \ | \ | \ | \ | \ |  |
| **CIPNAT** | 2011 | 36+14 | Paresthesia of limbs (numbness, tingling, cold sensitivity, nerve pain, muscle or joint aches) | \ | \ | Deep paresthesia of lower limbs (loss of balance) | \ | Muscle weakness of limbs (difficulty walking, picking up, holding, driving) | Fine motor coordination dysfunction (difficulty dressing, writing) | Autonomic dysfunction (interference with sexual activities, sleep) | Impact on mood and daily life (interference with work, hobbies, exercise, chores, relationships, enjoyment of life) | Numerical rating 0-10, containing symptom attribute (location, severity, frequency, disease time) |
|  |  |  | 56% | \ | \ | 8% | \ | 16% | 4% | 4% | 12% |  |
| **CAS-CIPN** | 2019 | 15 | Paresthesia of limbs (numbness, tingling, discomfort, dysethesia) | \ | \ | \ | \ | \ | Fine motor coordination dysfunction (difficulty turning over a sheet of paper, doing up bottons, snaps, and other fasteners) | \ | Attitude or mood to treatment or symptoms (depressed, anxious, happy, confidant, willpower) | Likert 0-4, described in the first person, describing personal feelings in detail |
|  |  |  | 60% | \ | \ | \ | \ | \ | 20% | \ | 20% |  |
| **TNAS**  ***v3.0*** | 2020 | 9 | Paresthesia of limbs (numbness, tingling, pain, hot or burning sensations, feelings of coldness) | \ | \ | Deep paresthesia of lower limbs (trouble with balance or falling) | \ | Muscle weakness of limbs (trouble walking) | Fine motor coordination dysfunction (difficulty using your hands or fingers) | \ | Impact on daily life (disturbed sleep) | Numerical rating 0-10 |
|  |  |  | 55.6% | \ | \ | 11.1% | \ | 11.1% | 11.1% | \ | 11.1% |  |
| Note: Item count number after “+” is the subscale item count | | | | | | | | | | | | |

| **Supplementary Table S5. Total variance explained** | | | | | | | | | | | |
| --- | --- | --- | --- | --- | --- | --- | --- | --- | --- | --- | --- |
| Component | Initial Eigenvalues | | | Extraction Sums of Squared Loadings | | | Rotation Sums of Squared Loadings | | | | |
|  | Total | % of Variance | Cumulative % | Total | % of Variance | Cumulative % | | Total | % of Variance | Cumulative % |  |
| 1 | 2.588 | 15.222 | 15.222 | 2.588 | 15.222 | 15.222 | | 1.909 | 11.230 | 11.230 |  |
| 2 | 2.060 | 12.117 | 27.339 | 2.060 | 12.117 | 27.339 | | 1.830 | 10.764 | 21.995 |  |
| 3 | 1.585 | 9.321 | 36.660 | 1.585 | 9.321 | 36.660 | | 1.802 | 10.600 | 32.594 |  |
| 4 | 1.424 | 8.379 | 45.039 | 1.424 | 8.379 | 45.039 | | 1.735 | 10.204 | 42.798 |  |
| 5 | 1.136 | 6.681 | 51.721 | 1.136 | 6.681 | 51.721 | | 1.419 | 8.349 | 51.147 |  |
| 6 | 1.098 | 6.460 | 58.180 | 1.098 | 6.460 | 58.180 | | 1.163 | 6.839 | 57.986 |  |
| 7 | 1.025 | 6.032 | 64.212 | 1.025 | 6.032 | 64.212 | | 1.058 | 6.225 | 64.212 |  |
| 8 | 0.924 | 5.437 | 69.648 |  |  |  | |  |  |  |  |
| 9 | 0.896 | 5.271 | 74.919 |  |  |  | |  |  |  |  |
| 10 | 0.791 | 4.653 | 79.572 |  |  |  | |  |  |  |  |
| 11 | 0.754 | 4.434 | 84.006 |  |  |  | |  |  |  |  |
| 12 | 0.714 | 4.202 | 88.208 |  |  |  | |  |  |  |  |
| 13 | 0.585 | 3.440 | 91.648 |  |  |  | |  |  |  |  |
| 14 | 0.553 | 3.250 | 94.899 |  |  |  | |  |  |  |  |
| 15 | 0.403 | 2.371 | 97.269 |  |  |  | |  |  |  |  |
| 16 | 0.257 | 1.510 | 98.779 |  |  |  | |  |  |  |  |
| 17 | 0.208 | 1.221 | 100.000 |  |  |  | |  |  |  |  |
