# Supplementary material for: Development and validation of the chemotherapy-induced peripheral neuropathy integrated assessment – oxaliplatin subscale: a prospective cohort study
Source: BMC Cancer. 2023 Nov 14;23:1109. doi: 10.1186/s12885-023-11541-7 (PMC10648311; doi:10.1186/s12885-023-11541-7)
Supplement: Supplementary file 2 — Additional file 2: Supplementary Fig. S1. Chemotherapy-induced Peripheral Neuropathy Integrated Assessment – Oxaliplatin Subscale (initial version). Scoring standard: "Not at all"=0, "A little bit"=1, "Quite a bit"=2, "Very much"=3. The symptom location was classified according to the Total Neuropathy Score (TNS). The symptom duration was classified according to the Levi’s scale. The influence was classified according to the National Cancer Institute Common Toxicity Criteria Adverse Events (NCI-CTCAE). Supplementary Fig. S2. Distributions of expert-cognitive clinical incidence and severity of symptoms affecting chemotherapy adjustment. The numbers in the column represent the number of experts who gave the evaluation corresponding to the color. (a) Expert-cognitive clinical incidence of oxaliplatin-induced neuropathic symptoms (scoring standard: Remote=0.25, Low=0.5, Moderate=0.75, High=1). (b) Expert-cognitive severity of oxaliplatin-induced neuropathic symptoms affecting chemotherapy adjustment (scoring standard: Mild=1, Moderate=0.66, Severe=0.33). Supplementary Fig. S3. Pre-testing item-level positive response rate (I-PRR) of the original version. Supplementary Fig. S4. Chemotherapy-induced Peripheral Neuropathy Integrated Assessment –Oxaliplatin Subscale (modified version). Scoring standard: "Not at all"=0, "A little bit"=1, "Quite a bit"=2, "Very much"=3. The symptom location was classified according to the Total Neuropathy Score (TNS). The symptom duration was classified according to the Levi’s scale. The influence was classified according to the National Cancer Institute Common Toxicity Criteria Adverse Events (NCI-CTCAE). [file 12885_2023_11541_MOESM2_ESM.docx]

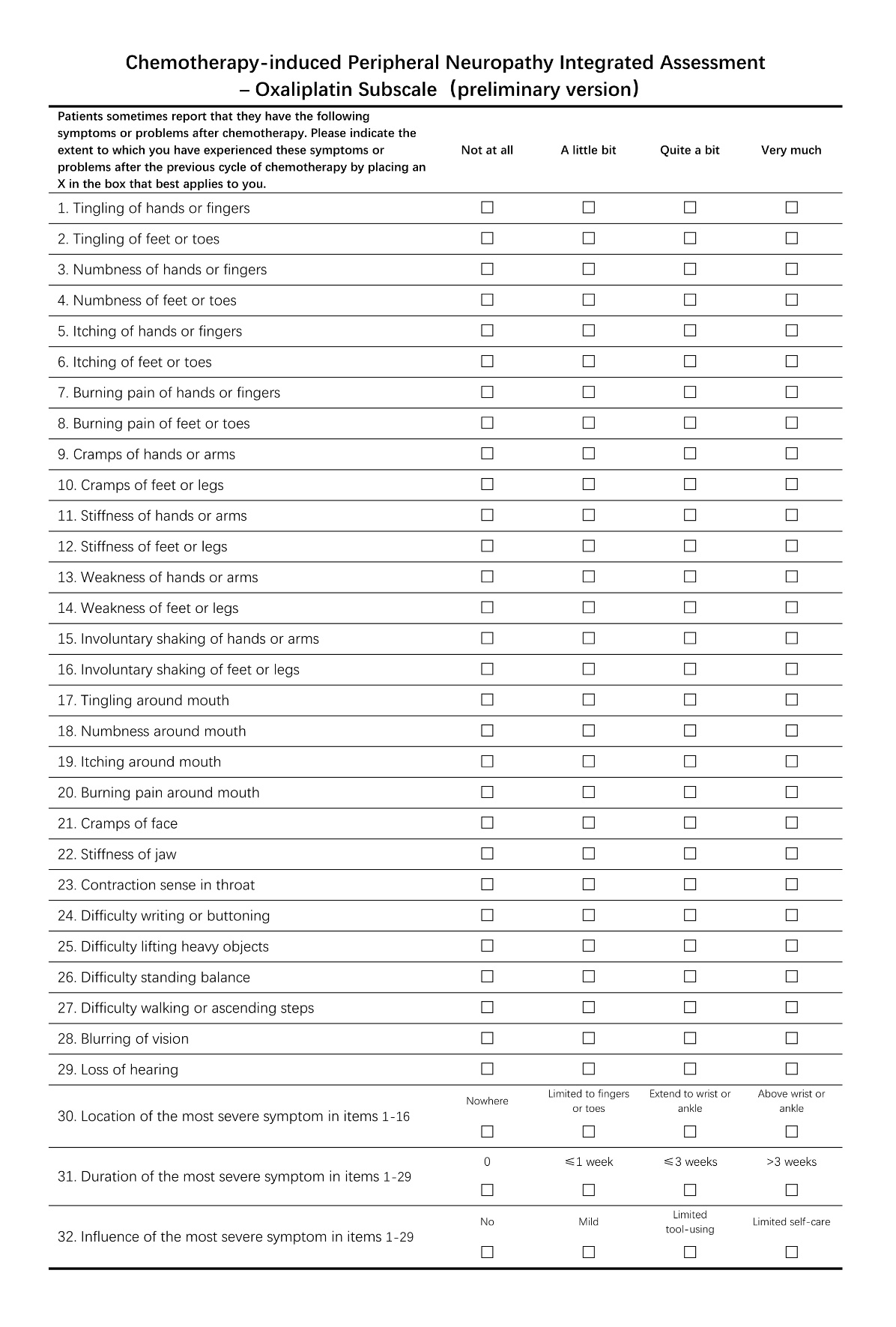
**Development and Validation of** **the Chemotherapy-induced Peripheral Neuropathy Integrated Assessment–Oxaliplatin Subscale**

**Supplementary Fig. S1** | Chemotherapy-induced Peripheral Neuropathy Integrated Assessment – Oxaliplatin Subscale (initial version).

Scoring standard: "Not at all"=0, "A little bit"=1, "Quite a bit"=2, "Very much"=3. The symptom location was classified according to the Total Neuropathy Score (TNS). The symptom duration was classified according to the Levi’s scale. The influence was classified according to the National Cancer Institute Common Toxicity Criteria Adverse Events (NCI-CTCAE).

**Supplementary Fig. S2** | Distributions of expert-cognitive clinical incidence and severity of symptoms affecting chemotherapy adjustment. The numbers in the column represent the number of experts who gave the evaluation corresponding to the color. **(a)** Expert-cognitive clinical incidence of oxaliplatin-induced neuropathic symptoms (scoring standard: Remote=0.25, Low=0.5, Moderate=0.75, High=1). **(b)** Expert-cognitive severity of oxaliplatin-induced neuropathic symptoms affecting chemotherapy adjustment (scoring standard: Mild=1, Moderate=0.66, Severe=0.33).


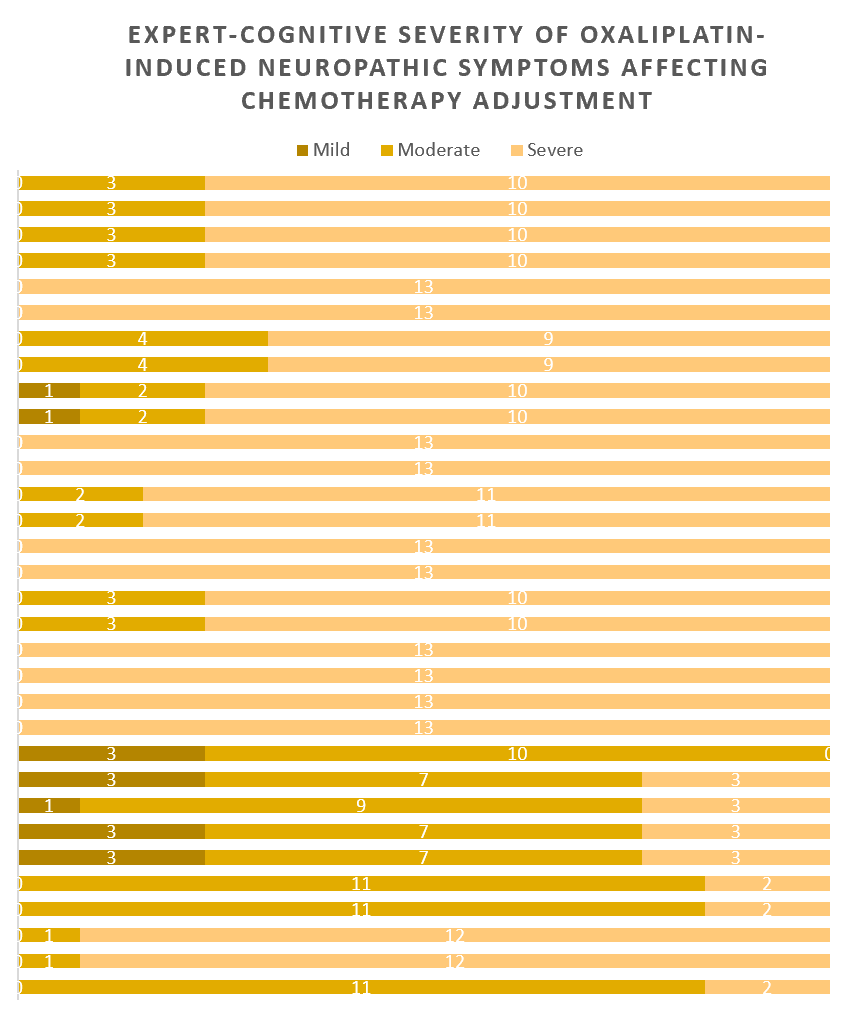

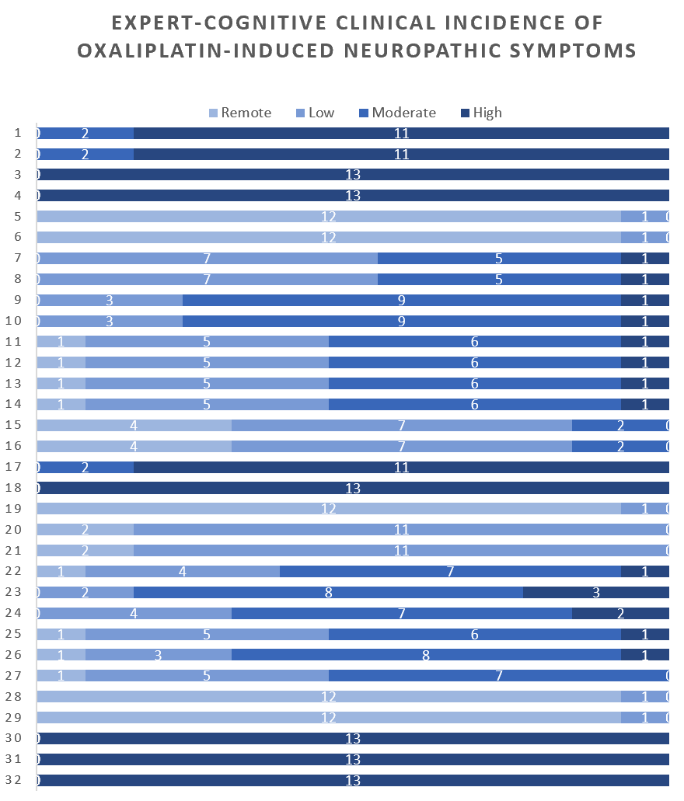


**a**

**b**

**Supplementary Fig. S3** | Pre-testing item-level positive response rate (I-PRR) of the original version


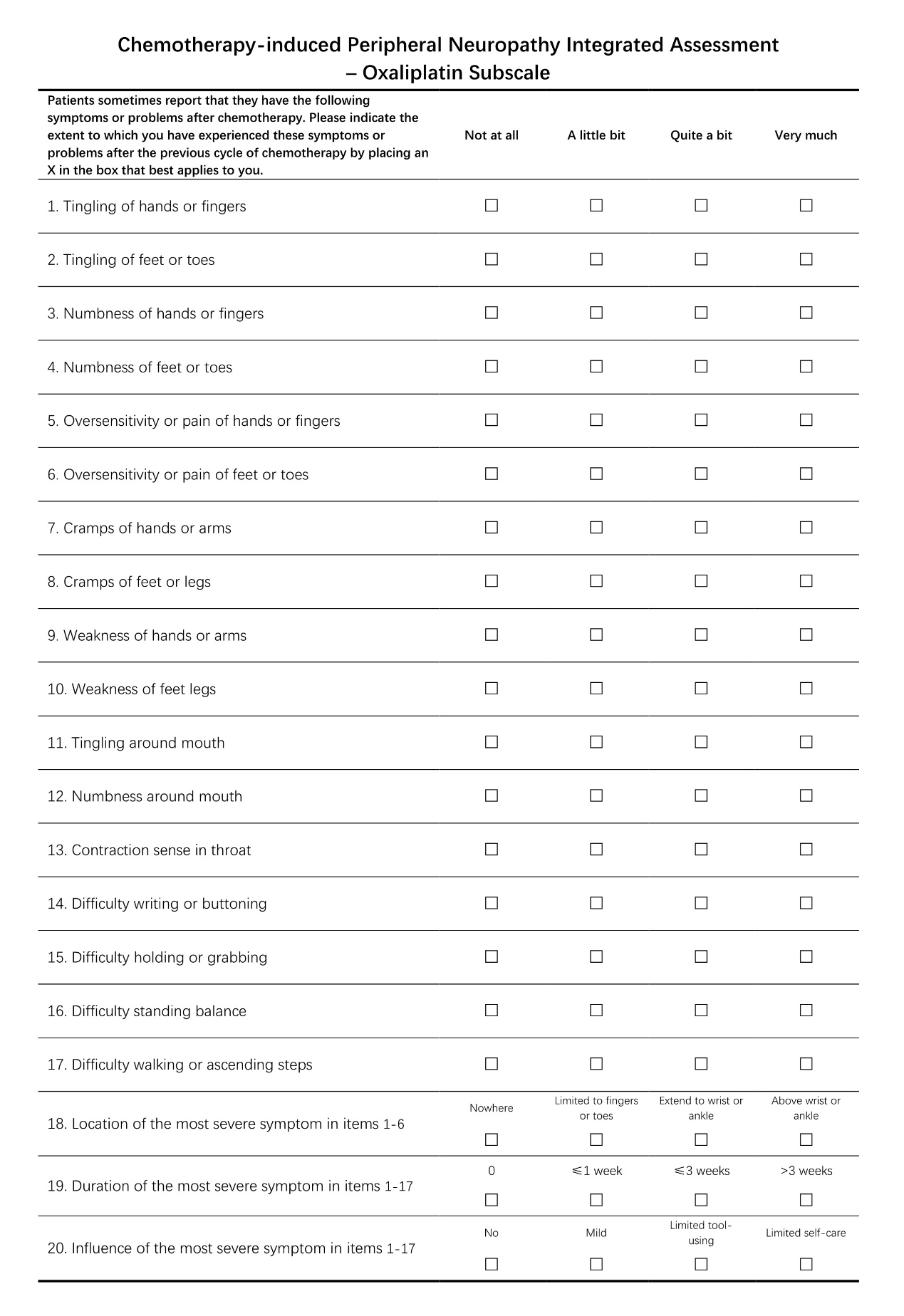
 **Supplementary Fig. S4** | Chemotherapy-induced Peripheral Neuropathy Integrated Assessment –Oxaliplatin Subscale (modified version)

Scoring standard: "Not at all"=0, "A little bit"=1, "Quite a bit"=2, "Very much"=3. The symptom location was classified according to the Total Neuropathy Score (TNS). The symptom duration was classified according to the Levi’s scale. The influence was classified according to the National Cancer Institute Common Toxicity Criteria Adverse Events (NCI-CTCAE).
